# Improving multiple sequence alignment by using better guide trees

**DOI:** 10.1186/1471-2105-16-S5-S4

**Published:** 2015-03-18

**Authors:** Qing Zhan, Yongtao Ye, Tak-Wah Lam, Siu-Ming Yiu, Yadong Wang, Hing-Fung Ting

**Affiliations:** 1 School of Computer Science and Technology, Harbin Institute of Technology, Harbin, China; 2 HKU-BGI Bioinformatics Algorithms & Core Technology Research Lab, Computer Science Department, University of Hong Kong, Hong Kong, China

**Keywords:** multiple sequence alignment, guide trees, phylogenetic trees

## Abstract

Progressive sequence alignment is one of the most commonly used method for multiple sequence alignment. Roughly speaking, the method first builds a guide tree, and then aligns the sequences progressively according to the topology of the tree. It is believed that guide trees are very important to progressive alignment; a better guide tree will give an alignment with higher accuracy. Recently, we have proposed an adaptive method for constructing guide trees. This paper studies the quality of the guide trees constructed by such method. Our study showed that our adaptive method can be used to improve the accuracy of many different progressive MSA tools. In fact, we give evidences showing that the guide trees constructed by the adaptive method are among the best.

## Introduction

Multiple sequence alignment is a basic task in Bioinformatics and has many applications in biological analyses such as phylogenetic inferencing and protein 3D structure prediction. The progressive alignment method [1] is one of the most commonly used methods for multiple sequence alignment. Roughly speaking, the method first constructs a guide tree that is supposed to capture the phylogenetic relationship of the input sequences, and then aligns the sequences progressively according to the topology of the guide tree such that more related sequences are aligned first and the less related ones are aligned later.

Recently, we have proposed an adaptive approach for progressive multiple sequence alignment[2]. We observed that for different sequence families with different similarities, their alignments usually have different characteristics and structural properties, and by using some reliable measure to estimate the similarity of the inputs, we may exploit the corresponding properties to help generate better alignments. To estimate the similarity, we proposed to use the average percent identity, which is defined as follows. For any two sequences, the percent identity of these two sequences is defined to be

$$\text{PID}=\frac{N_{\text{Identity}}}{L_{\text{Alignment}}}$$

where *N*_Identity _is the number of identities in the optimal pairwise alignment of the two sequences, and *L*_Alignment _is the length of this alignment. The average percent identity $\bar{\text{PID}}$ of the input sequences is the average of the PIDs over every pair of the sequences. In [2], we noted that if $\bar{\text{PID}}$ is greater than 40%, the input sequences are very similar, and we showed how to exploit the properties of similar sequences and align the sequences globally. If $\bar{\text{PID}}$ is between 25% and 40%, the input are moderately similar, and we can exploit the corresponding properties to align them locally. For input below 25%, we do not know which alignment methods is better; hence we suggested trying different methods (e.g., using global alignment methods as well as local alignment methods) and using their consensus to determine the final alignment.

To test the effectiveness of our idea, we developed a software tool called GLProbs, which implements our adaptive approach for multiple sequence alignment. We have done extensive testings and empirical comparisons for GLProbs, and the results showed that GLProbs has significantly better accuracy than a dozen of other leading MSA tools (see [2] for more details).

In this paper, we study why GLProbs can achieve such a high accuracy, and exploit ways to further improve the software tool. In particular, we are interested in finding out the impact of the adaptive guide tree construction method used in GLProbs. This also leads us to study the following fundamental question:

Are guide trees really important to obtain high quality multiple sequence alignments, and if yes, how to construct the best guide trees.

We note that there are already studies suggesting that guide trees are important. For example, Penn *et al*.[3] showed that uncertainties in the guide tree lead to a major source of alignment uncertainty, and Capella-Gutierrez and Gabaldon[4] showed that most gaps are inserted in patterns that follow the guide tree.

To study the guide trees of GLProbs, we have done the following tests.

First, we modified GLProbs to GLProbs-Random in which the adaptive guide tree construction step of GLProbs was replaced by a step that just generates a random guide tree. Then we compared the performance of GLProbs and GLProbs-Random empirically.

Second, we modified GLProbs to a new tool GLProbs-Reference and compared their performance of aligning families of protein sequences whose correct multiple sequence alignments are generally agreed by the biologists. The modification done to get GLProbs-Reference is that the guide tree generated by GLProbs is replaced by the phylogenetic tree constructed as follows: Based on the known correct alignment of the input sequences we construct their phylogenetic trees using the maximum-likelihood method [5], and then use these phylogenetic trees as the guide trees. Intuitively these phylogenetic trees should be the best guide trees for the alignments. The aim of this test is to find out whether the guide trees constructed by the adaptive method are competitive among the best.

Finally, we study whether the adaptive guide tree construction method of GLProbs can bring similar improvement to other MSA tools. We have modified five leading multiple sequence alignment tools, namely MSArobs [6], Probalign [7], Prob-Cons [8], T-Coffee [9], ClustalW [10], by replacing their original guide trees construction steps with the adaptive guide tree construction step, and keeping other steps intact. Then we compare their performance on aligning protein sequence families obtained from three popular benchmark datasets.

We will detail the results of our tests in Sections 2, 3 and 4. Below, we summarise our conclusions.

• For sequences with high similarity, the guide tree construction method is not critical; many reasonable methods can generate good enough guide trees leading to satisfactory alignments.

• For sequences with moderate similarity, better guide trees are very important for generating good alignments. Our study showed that the guide trees constructed by the adaptive method of GLProbs are usually among the best, and they can be used to improve the performance of other MSA tools.

• For sequences with very low similarity, the adaptive guide tree construction method can also improve the accuracy of other MSA tools; in fact, the improvements are larger than those obtained for other more similar sequences. However, the accuracy of these alignments is still very low. We found that for sequences with very low similarity, it is very difficult to generate good guide trees, and using a bad guide tree will have serious detrimental effect on the quality of the resulting alignment. For these sequences, we suggest using other methods, such as the non-progressive alignment method, that do not rely on guide trees for generating better alignments.

## Comparing adaptive guide trees with random trees

As aforementioned, the progressive multiple sequence alignment method needs to construct a guide tree to determine the order of the progressive alignments. Intuitively, the accuracy of the alignments depends much on the quality of the guide trees; if the aligned orders are wrong, the accuracy may be low.

**Figure 1:**
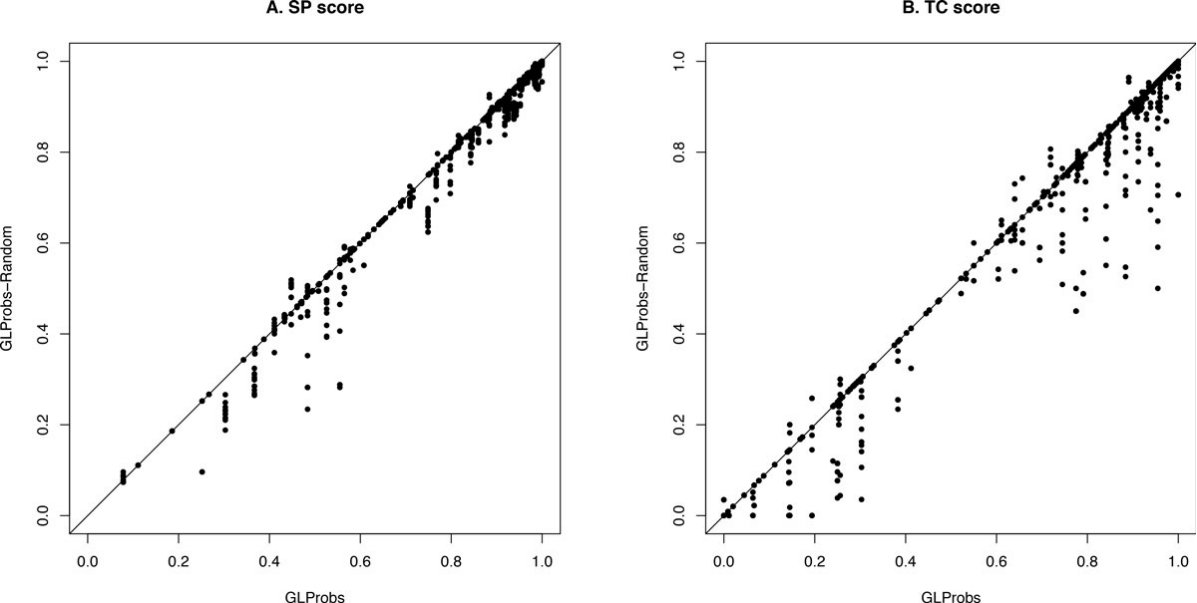
**GLProbs vs GLProbs-Random on OXBench in term of SP and TC scores**.

To confirm this intuition, we have modified GLProbs to GLProbs-Random, which replaces the guide tree constructed in GLProbs by a random guide tree. We have used them to align protein sequences families obtained from the benchmark database OXBench. Figure 1 shows their alignments' sum-of-pairs (SP) scores and total column (TC) scores, two of the most commonly used scores for measuring the quality of MSA. Each dot (*x, y*) in the figure shows the scores obtained by GLProbs and GLProbs-Random for one testing sample, where *x *is the score obtained by GLProbs and *y *by GLProbs-Random. Unsurprisingly, we note that most points are below the diagonals, which means GLProbs outperformed GLProbs-Random. This confirms the importance of guide trees. However, it is interesting to observe that there are also many points above the diagonals, which means that for these inputs, random guide trees are better than the guide trees elaborately generated by GLProbs. After a careful study of the inputs, we found that most of these inputs have low similarities. We believe that to generate better alignments for these inputs, we should abandon the progressive method, and try other methods such as the non-progressive alignment method [11], that do not rely on guide trees to generate their alignments.

## Using adaptive guide trees to improve other leading MSA tools

To study whether the adaptive guide tree construction method of GLProbs can improve the accuracy of other MSA tools, we have applied it to five leading multiple sequence alignment tools: MSArobs [6], Probalign [7], ProbCons [8], T-Coffee [9] and ClustalW [10], by modifying these tools so that they used the adaptive guides trees constructed by GLProbs. We note that these five tools have their own special features. ClustalW is among the first tools using progressive alignment, and has become one of the most popular MSA tools since its release in 1994. MSAProbs, Probalign and ProbCons apply the consistency-based method to improve the accuracy of the progressive alignment. T-Coffee provides a simple and flexible means of producing multiple sequence alignments by using heterogeneous data sources given by a library of global and local pairwise alignments.

In our tests, we used samples from three popular benchmark datasets, namely BAliBASE [12], OXBench [13], and SABmark [14]. In particular, we use the two subsets RV11 and RV12 in BALiBASE, where RV11 contains distant sequences with < 20% identity while RV12 consists of medium to divergent sequences with identities between 20% and 40%. For SABmark we used its two subsets: Twilight Zone and Superfamily. Twilight Zone represents different SCOP folds subsets, where each subset contains sequences with no more than 25% identity. Superfamily contains different SCOP superfamilies, which have no more than 50% identity. For OXBench, the families of sequences we used ranging from 0% to 100% similarity.

Table 1 shows the average SP and TC scores obtained from the original alignment tools (listed in the columns labeled with "Original"), and those obtained by the modified tools, in which their guide trees are replaced by the ones constructed by the adaptive method of GLProbs (listed in the columns labeled with "Adpative"). Note that the average SP scores of all of the five aligners with guide tree generated by the adaptive method outperform those generated by original aligners on all of the three benchmarks. For TC scores, we can see that using guide tree generated by the adaptive method can improve the average score in most cases.

**Table 1:** Mean SP and TC scores on BAliBASE, OXBench and SABmark

		SP		TC	
		**Adaptive**	**Original**	**Adaptive**	**Original**

BAliBASE	ClustalW	**71.277**	69.578	**51.865**	49.121
	MSAProbs	**82.683**	82.370	**67.449**	67.274
	Probalign	**83.153**	82.991	**68.054**	67.691
	ProbCons	**82.007**	81.541	**66.284**	65.618
	T-Coffee	**81.646**	80.759	**66.457**	64.894

OXBench	ClustalW	**89.833**	89.446	**80.409**	80.189
	MSAProbs	**90.092**	90.062	81.696	**81.703**
	Probalign	**89.997**	89.966	81.642	**81.680**
	ProbCons	**89.719**	89.680	**80.895**	80.880
	T-Coffee	**89.534**	89.519	**80.680**	80.513

SABmark	ClustalW	**52.472**	51.957	**31.927**	31.495
	MSAProbs	**60.280**	60.245	39.946	**40.044**
	Probalign	**59.666**	59.532	**38.941**	38.626
	ProbCons	**59.826**	59.690	**39.437**	39.166
	T-Coffee	**59.377**	59.158	39.291	**39.597**

Average SP score and average TC score of the alignments on the three benchmarks generated by the five aligners with guide tree generated by adaptive method and by aligners' own. Rows show the average sum of pairs scores (SP) and total column scores (TC) multiplied by 100. The best results in each pair are shown in bold.

We also divided OXBench's input families into four categories according to their similarities. For example, the "0%-20%" category contains the families that the similarities of which are between 0% and 20%. Category "0%-100%" contains all of the families. In Table 2 it can be seen that the adaptive method can improve most aligners in most cases, especially in the low similarity categories.

**Table 2:** Mean SP and TC scores on OXBench

		ClustalW		MSAProbs		Probalign		ProbCons		T-Coffee	
		**Adaptive**	**Original**	**Adaptive**	**Original**	**Adaptive**	**Original**	**Adaptive**	**Original**	**Adaptive**	**Original**

SP	0%-100%	**89.833**	89.446	**90.092**	90.062	**89.997**	89.966	**89.719**	89.680	**89.534**	89.519
	
	0%-20%	**48.219**	42.944	**45.140**	44.840	**44.308**	43.576	**45.390**	44.140	**44.883**	43.818
	20%-40%	**77.364**	77.061	**77.874**	77.839	**77.261**	77.259	77.026	**77.049**	76.545	**76.667**
	40%-70%	**93.982**	93.778	**94.569**	94.542	**94.691**	94.688	94.220	**94.233**	**94.146**	94.139
	70%-100%	**99.274**	99.236	99.25	**99.260**	**99.319**	**99.319**	**99.138**	**99.138**	99.055	**99.070**

TC	0%-100%	**80.409**	80.189	81.696	**81.703**	81.642	**81.680**	**80.895**	80.880	**80.680**	80.513
	
	0%-20%	**20.883**	18.233	**22.078**	**22.078**	20.462	**20.518**	**20.988**	20.301	**19.663**	19.108
	20%-40%	**57.570**	57.255	**59.863**	59.817	59.107	**59.379**	58.277	**58.340**	**57.968**	57.938
	40%-70%	**86.403**	86.363	**87.966**	87.933	**88.255**	88.204	**87.309**	87.307	**87.212**	86.941
	70%-100%	**98.005**	97.913	97.972	**98.093**	**98.135**	**98.135**	**97.616**	**97.616**	**97.442**	97.402

Average SP score and average TC score of the alignments on OXBench generated by the five aligners with guide tree generated by adaptive method and by aligners' own. Rows show the average sum of pairs scores (SP) and total column scores (TC) multiplied by 100. The best results in each pair are shown in bold.

Table 3 and Table 4 show the results on SABmark and BAliBASE, in which the results are divided into two categories according to the similarity of the input families. All of the aligners have improvement in the low similarity "0%-30%" category, except the average TC score of T_Coffee's alignments on SABmark.

**Table 3:** Mean SP and TC scores on BAliBASE

		ClustalW		MSAProbs		Probalign		ProbCons		T-Coffee	
		**Adaptive**	**Original**	**Adaptive**	**Original**	**Adaptive**	**Original**	**Adaptive**	**Original**	**Adaptive**	**Original**

SP	0%-60%	**71.277**	69.578	**82.683**	82.370	**83.153**	82.991	**82.007**	81.541	**81.646**	80.759
	
	0%-30%	**52.878**	51.573	**69.251**	68.584	**70.183**	69.826	**68.289**	67.300	**67.543**	65.676
	30%-60%	**86.404**	84.382	**93.727**	93.704	**93.818**	93.816	**93.287**	93.251	**93.242**	93.160

TC	0%-60%	**51.865**	49.121	**67.449**	67.274	**68.054**	67.691	**66.284**	65.618	**66.457**	64.894
	
	0%-30%	**26.754**	26.014	**46.546**	46.143	**47.989**	47.178	**44.776**	43.332	**45.122**	42.346
	30%-60%	**72.511**	68.120	84.636	**84.649**	84.551	**84.558**	**83.969**	83.942	**84.000**	83.433

Average SP score and average TC score of the alignments on BaliBASE3 generated by the five aligners with guide tree generated by adaptive method and by aligners' own. Rows show the average sum of pairs scores (SP) and total column scores (TC) multiplied by 100. The best results in each pair are shown in bold.

**Table 4:** Mean SP and TC scores on SABmark

		ClustalW		MSAProbs		Probalign		ProbCons		T-Coffee	
		**Adaptive**	**Original**	**Adaptive**	**Original**	**Adaptive**	**Original**	**Adaptive**	**Original**	**Adaptive**	**Original**

SP	0%-60%	**52.472**	51.957	**60.280**	60.245	**59.666**	59.532	**59.826**	59.690	**59.377**	59.158
	
	0%-30%	**44.504**	43.907	**52.081**	51.996	**51.251**	51.161	**51.601**	51.459	**51.266**	50.799
	30%-60%	**81.954**	81.739	90.616	**90.766**	**90.802**	90.507	**90.258**	90.146	89.388	**90.087**

TC	0%-60%	**31.927**	31.495	39.946	**40.044**	**38.941**	38.626	**39.437**	39.166	39.291	**39.597**
	
	0%-30%	**22.398**	22.020	**29.132**	29.043	**27.894**	27.702	**28.559**	28.198	28.518	**28.599**
	30%-60%	**67.183**	66.551	79.954	**80.748**	**79.816**	79.044	79.687	**79.747**	79.151	**80.291**

Average SP score and average TC score of the alignments on SABmark generated by the five aligners with guide tree generated by adaptive method and by aligners' own. Rows show the average sum of pairs scores (SP) and total column scores (TC) multiplied by 100. The best results in each pair are shown in bold.

## Comparing adaptive guide trees with reference guide trees

**Figure 2:**
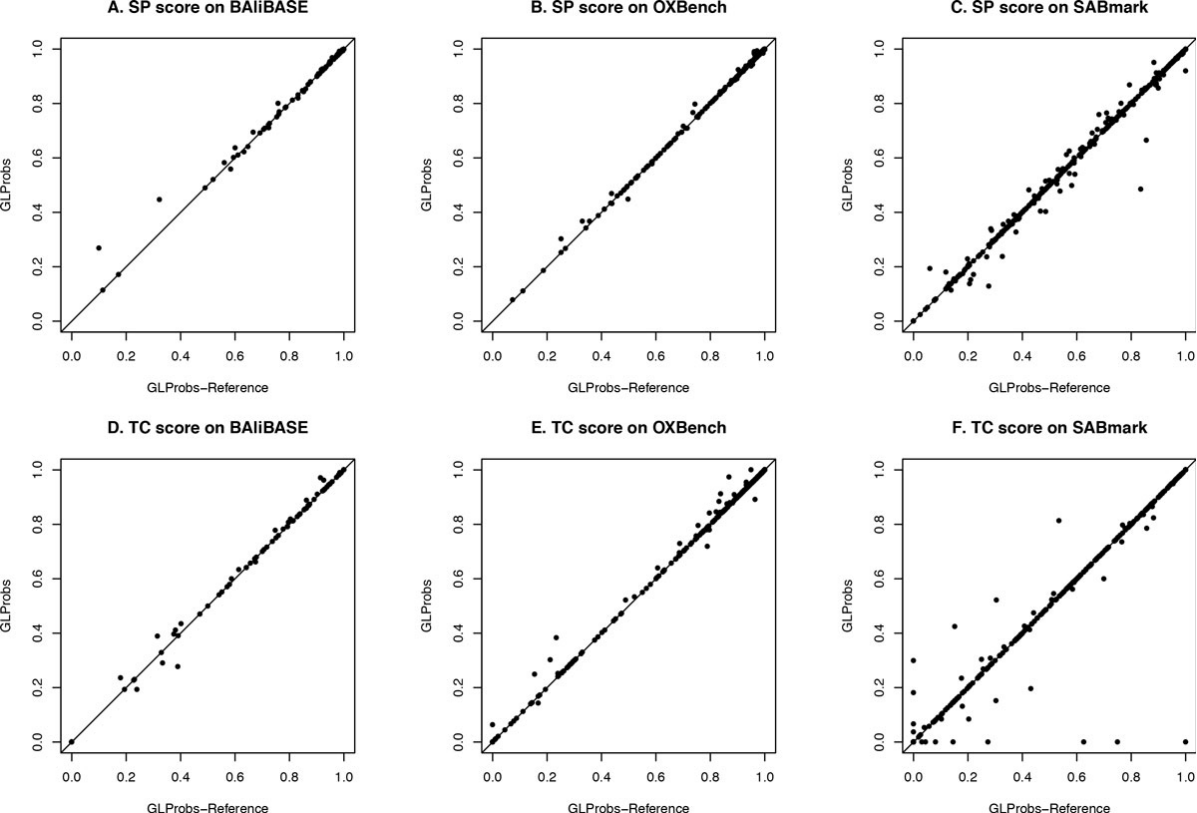
**GLProbs vs GLProbs-Reference on BAliBASE, OXBench, SABmark**. Dots above diagonal represent GLProbs outperformed GLProbs-Reference.

To compare the adaptive guide trees with the best ones, we modified GLProbs to GLProbs-Reference, in which the guide tree generated by GLProbs is replaced by the phylogenetic tree constructed by applying the maximum-likelihood method [5] on the correct MSA of the input sequences. Figure 2 compares the SP and TC scores of the alignments constructed by GLProbs and GLProbs-Reference for the sequence families obtained from the three benchmark databases BAliBASE, OXBench and SABmark. The figure shows that most points located around the diagonal, which suggests the performances of using the reference (best) guide tree and that generated by the adaptive scheme are similar.

## Competing interests

The authors declare that they have no competing interests.

## Authors' contributions

HFT conceived the project, QZ, YY and HFT designed the project, QZ, YY implemented the project, TWL, SMY and YW provided feedbacks on the implementation.

## Declarations

Publication costs for this article were funded by the authors' departmental resources.

This article has been published as part of *BMC Bioinformatics *Volume 16 Supplement 5, 2015: Selected articles from the 10th International Symposium on Bioinformatics Research and Applications (ISBRA-14): Bioinformatics. The full contents of the supplement are available online at http://www.biomedcentral.com/bmcbioinformatics/supplements/16/S5.
